# History of Falls, Dementia, Lower Education Levels, Mobility Limitations, and Aging Are Risk Factors for Falls among the Community-Dwelling Elderly: A Cohort Study

**DOI:** 10.3390/ijerph18179356

**Published:** 2021-09-04

**Authors:** Yan-Yuh Lee, Chien-Liang Chen, I-Chen Lee, I-Ching Lee, Nai-Ching Chen

**Affiliations:** 1 Department of Physical Medicine and Rehabilitation, Kaohsiung Chang Gung Memorial Hospital, Kaohsiung 833, Taiwan; b9005009@cgmh.org.tw; 2 Division of Nephrology, Kaohsiung Veterans General Hospital, Kaohsiung 813, Taiwan; cclchen1@vghks.gov.tw; 3 Department of Medicine, National Yang-Ming University School of Medicine, Taipei 112, Taiwan; 4 Department of Healthcare Administration and Medical Informatics, Kaohsiung Medical University, Kaohsiung 807, Taiwan; ichen@kmu.edu.tw; 5 Ever Blessing Clinic, Kaohsiung 804, Taiwan; ching.everjoy@gmail.com; 6 Department of Neurology, Kaohsiung Chang Gung Memorial Hospital, Chang Gung University College of Medicine, Kaohsiung 833, Taiwan

**Keywords:** previous falling event, reduced cognition, Alzheimer dementia, elderly, fall

## Abstract

Background: Falling is a serious issue among elderly community dwellers, often resulting in disability. We aimed to investigate the risk factors for falls among elderly community dwellers. Methods: We recruited 232 participants from multiple community learning and care centers, who provided their information through questionnaires. They were divided into two groups, according to their falling events after a 1-year follow-up. Univariate and multivariate logistic regressions were used for statistical analysis. Results: A total of 64 participants reported a fall at the 1-year follow-up. The falling group comprised older and single people with lower education levels, higher rates of dementia, a history of falls, lower scores on the Mini-Mental State Examination, and more disability functions when compared to the non-falling group (all *p* < 0.05). The regression model showed that a history of falls (OR: 62.011; *p* < 0.0001), lower education levels (OR: 4.088; *p* = 0.039), mild dementia (OR: 20.729; *p* = 0.028), older age (OR: 1.176; *p* < 0.0001), walking for 300 m (OR: 4.153; *p* = 0.030), and running for 30 m (OR: 3.402; *p* = 0.015) were 1-year risk factors for falls. Conclusion: A history of falling, low education levels, aging, mild dementia, and certain mobility limitations were strong risk factors for future falling accidents in elderly Taiwanese community dwellers.

## 1. Introduction

Falling is a common health threat, with a high prevalence, of approximately 20–45% [1,2,3]. The prevalence of falls among the elderly in Taiwan is around 16.3% to 21.3% [4,5], which is similar to that observed in other countries around the world. Moreover, falling is a major cause of disability and death among the elderly and is related to multiple public health problems worldwide [3]. Fall-related injuries, such as hip fractures, traumatic brain injuries, and intracranial hemorrhage, are related to disabilities and complications, including pressure sores and infections, and, eventually, increased morbidity and mortality; hence, the consequences of falls are serious in the older adult population [6,7,8,9,10]. Therefore, it is important to identify the risks for falls among the elderly so that they can benefit from fall-prevention programs.

The risks for falls among elderly community dwellers are multifactorial, including the environment, a history of falling, the individual’s psychology, mobility, sensation, cognition, and psychosocial status, as well as polypharmacy [11,12]. In this group of people, aging is a key factor related to falling, as fall-related mortality rates increase exponentially with age, with the greatest increase after the age of 80 years [3,13]. This is because age-related conditions, including physical frailty, immobility, reduced functional capacity, and medical conditions contribute to the risk of falls [3,13,14,15,16]. Therefore, investigating the risk factors for falls among the elderly is often challenging because the complex aging-related conditions and the interaction among the risk factors make it difficult to estimate the extent of the impact of certain risk factors [17].

In elderly community dwellers, decreased and impaired mobility has been noted as a risk factor for falls [18,19]. A similar report investigated a facility and found that impaired mobility was associated with falling [20]. Decreased mobility, poor physical performance, slow gait speed, and increased mortality are common in the elderly; these characteristics are defined as sarcopenia, due to the associated loss of muscle strength and mass [21]. Therefore, it is crucial to evaluate the mobility of elderly community dwellers in order to prevent falls.

A self-administered questionnaire was developed in Taiwan [22] to evaluate mobility and fitness. Considering the high prevalence of falls, a user-friendly, self-administrated screening tool for the assessment of risk factors for falls could be used to improve alertness and prevent falling. The aim of this study was to investigate the risk factors for falls among elderly community dwellers on a longitudinal scale and to evaluate the relationship between falling and its risk factors, including mobility limitations, cognitive function, and other demographic factors.

## 2. Materials and Methods

### 2.1. Study Design

This was an observational cohort study. The study hospital, Kaohsiung Chang Gung Memorial Hospital, is a tertiary medical center in southern Taiwan. This study was conducted in accordance with the Declaration of Helsinki and was approved by the Institutional Review Board of Chang Gung Memorial Hospital (CGMH-IRB No 201801933B0C501). All participants provided informed consent.

### 2.2. Subjects

We enrolled participants via advertisements on bulletin boards in community centers, community colleges, and local dementia community centers. A total of 232 participants were recruited for this study. The inclusion criteria were people >55 years of age who were socially active, able to answer the questionnaire, able to complete the examination, and able to provide signed informed consent. The excluded participants had poor functional status (for example, they needed a cane, a walker, or a wheelchair for mobility).

### 2.3. Demographic Data and Falling Events

The demographic data of the patients, including their age, sex, marital status, years of education, and exercise habits, were checked by the case manager using a formal questionnaire. For disease comorbidities, we recorded the patients’ history of systemic diseases, including diabetes mellitus, hypertension, hyperlipidemia, anemia, and stroke. Falling within the past year was recorded as recent fall history. One year after enrollment, the participants were re-evaluated and asked if there were falling events during this period.

Exercise habits were defined using our questionnaire. We surveyed exercise habits according to a previous study [23]. The patients were asked the following question during the interview: “Do you practice sports or physical exercise sufficient to produce sweating or shortness of breath?” Those who answered with a “yes” were defined as having exercise habits. They were also divided into two groups according to exercise frequency: <3 per week and ≥3 per week.

### 2.4. Cognitive Testing

We used a comprehensive battery of tests to assess participants’ cognitive abilities and the Mini-Mental State Examination (MMSE) [24] to assess their general intellectual function. Cognitive severity was evaluated using the Clinical Dementia Rating (CDR) Scale [25,26]. Both were administered by a trained neuropsychologist at the dementia integrated outpatient clinic. Among all participants, those with mild dementia (defined as CDR 1) or questionable dementia (defined as CDR 0.5) were recruited. Mild dementia indicated that the subject had moderate memory loss, especially for recent events, along with some impairment in other cognitive domains [26]. Subjects with questionable dementia showed slight forgetfulness, along with some slight impairment in other cognitive domains [26].

### 2.5. Mobility Limitation Questionnaire

The self-administrated questionnaire [22,27] included 10 simple questions about physical limitations by level of difficulty, which were asked by our case manager. They referred to: (1) standing continuously for 15 min; (2) standing continuously for 2 h; (3) sitting for 2 h; (4) squatting; (5) raising both hands over the head; (6) grasping/turning objects without fingers; (7) lifting 12 kg; (8) walking 300 m; (9) climbing three flights of stairs; and (10) running 30 m.

### 2.6. Statistical Analysis

Statistical analyses were performed using SPSS software (version 22; IBM, Inc., Chicago, IL, USA). All parameters were expressed as the mean ± standard deviation or a number (percentage). The participants were categorized into the falling group if they fell within 1 year of follow-up; otherwise, they were categorized into the non-falling group. A chi-squared test was used to analyze categorical variables, such as sex, chronic disease, education, and mobility limitations, between the falling and non-falling groups. An independent *t*-test was used to analyze continuous variables, such as age. Univariate analysis using chi-squared tests was used to analyze the association between falls. We chose factors with significant differences, including age, MMSE, marital status, dementia, and history of falls, and the factors with significant differences on the mobility limitation questionnaire, including standing continuously for 15 min, standing continuously for 2 h, squatting, lifting 12 kg, walking 300 m, climbing three flights of stairs, and running 30 m, for a binary logistic regression analysis. Multiple binary logistic regression was used to determine the factors associated with falls among elderly community dwellers. The statistical significance was set at *p* < 0.05.

## 3. Results

### 3.1. Characteristics of Study Participants

Older adults (172 female/60 males) aged between 55 and 93 years (mean age = 70.5 years; SD = 9.2) were recruited for analysis. The mean number of years of education was 10.0 years, with an SD of 3.8. Of these participants, 41.8% were single or widowed and 58.2% were married. The mean MMSE score of the 232 subjects was 26.3, with an SD of 4.2 (Table 1).

Sixty-four participants reported falling 1 year after enrollment. The age of the falling group (76.9 ± 7.5) was significantly higher than that of the non-falling group (68.0 ± 8.7) (*p* < 0.001). The falling group had significantly lower education levels (*p* < 0.001) and more widowed or single individuals (*p* = 0.014) than the non-falling group. The falling group had a significantly higher history of falls than the non-falling group (71.9% vs. 11.9%; *p* < 0.001). There was a significant difference between the falling and non-falling groups in terms of dementia (*p* = 0.012). The 14 participants diagnosed with mild dementia were all suffering from the Alzheimer’s disease type. The falling group had significantly lower MMSE scores than the non-falling group (24.7 ± 4.7, 27.0 ± 3.8; *p* = 0.001).

There was no significant difference between the falling and non-falling groups in terms of sex, exercise frequency, hypertension, hyperlipidemia, diabetes mellitus, stroke, anemia, or BMI. None of the participants had reported a history of Parkinson’s disease or another neurodegenerative disease that could confound the results. The detailed clinical characteristics and demographic data of all the participants are shown in Table 1.

### 3.2. Mobility Limitations of Study Participants

Table 2 reveals the differences in mobility limitations between the two groups. There was a significant difference between the two groups, with the non-falling group having a greater percentage of participants facing no limitation in standing continuously for 15 min (95.8% vs. 84.5%; *p* = 0.003), standing continuously for 2 h (54.2% vs. 29.7%; *p* = 0.001), squatting (61.9% vs. 37.5%; *p* = 0.001), lifting 12 kg (63.1% vs. 46.9%; *p* = 0.025), walking 300 m (65.5% vs. 31.3%; *p* < 0.001), climbing three flights of stairs (82.7% vs. 60.9%; *p* < 0.001), and running 30 m (65.5% vs. 31.3%; *p* < 0.001).

### 3.3. Risk Factors of Falling among Elderly Community Dwellers

As shown in Table 3, we found that a lower education level (≤6 years) was a risk factor for falls (odds ratio (OR) = 4.088; 95% confidence interval (CI) = 1.075–15.549; *p* = 0.039) after adjusting for age, MMSE, dementia, marital status, and history of falls compared a level of education >7 years. Older participants were also at a higher risk of falls (OR = 1.176; 95% CI = 1.101–1.256; *p* < 0.0001). Subjects with mild dementia (OR = 20.729; 95% CI = 1.376–312.195; *p* = 0.028) were at a significantly higher risk for falls than those with normal cognition or questionable dementia. Additionally, subjects with a history of falls (OR = 62.011; 95% CI = 19.090–201.44; *p* < 0.0001) were at a significantly higher risk for falls than those without a history of falls. Mobility limitations, including walking 300 m (OR = 4.153; 95% CI = 1.144–15.077; *p* = 0.03) and running 30 m (OR = 3.402; 95% CI = 1.267–9.131; *p* = 0.015) were associated with future falling events.

## 4. Discussion

The study participants were recruited from multiple community activity centers, demonstrating the ecological validity of the study. The findings of the present study were as follows: (1) community-dwelling and socially active subjects with a history of falling had a significantly increased risk of future falls compared with those without a history of falling; (2) subjects with greater overall severity measured by CDR at enrollment were significantly more likely to have future falling events; (3) older subjects with ≤6 years of education were at a significantly higher for falls in the future; and (4) mobility limitations, including walking and running, were associated with future falling events.

We did not exclude patients with a history of falls, but we excluded participants who needed an assistance device to move. However, a history of falls was the strongest predictor of future falls in our study, and this result is in agreement with those of previous studies [13,17]. A history of falls is crucial because it may result in a fear of falling, and fear may prevent older adults from engaging in relatively dangerous activities [28]. In the short term, the fear of falling may seem to reduce the occurrence of falls among older adults as they avoid certain activities, and may protect older adults from immediate danger. However, in the long term, it may limit the ability of older adults to perform daily activities, resulting in a decline in physical function [29]. As a result, identifying the fall history and any mobility limitations have the same importance in elderly community dwellers in terms of both short-term and long-term effects. In our study, mobility limitations in walking and running were predictive of future falling events, and these mobility limitations represented the lack of endurance or strength of the lower extremities, which are both associated with falling [13]. Therefore, we should also pay attention to older adults with a history of falls to improve their physical fitness and reduce the incidence of falls. A history of falls and mobility limitations are equally important.

In addition to daily activities, social activity can also improve physical and mental health and reduce the risk of falls [30,31]. We included participants from community centers, community colleges, and local dementia community centers. They were all socially active, but those with lower education levels had a higher number of falls in our study within the socially active population. A previous 4-year cohort study of a population aged 45 years and over also found that participants with fall incidents had lower education levels [30]. Elderly people with lower education levels may have difficulty grasping fall-prevention information [32]; therefore, a fall-prevention program is warranted in this group of people.

Dementia is a risk factor; individuals with mild dementia had a higher chance of falling compared to those with normal cognition and questionable dementia in our study. Generally, it is well accepted that dementia is associated with falling [11], and impaired attention and executive function are the main causes of falls among patients with dementia [13]. There are two possible explanations for our results indicating that patients with questionable dementia do not have a higher risk of falling compared to cognitively intact elderly individuals. First, patients with questionable dementia may already have adequate medical advice, education on therapeutic exercise, and even a fall-prevention program, which prevents them from falling [33]. Second, the healthy controls may have subclinical impaired attention or develop dysfunction of attention and executive function in the follow-up period, which might increase their risk of falling and confound the results [34]. Therefore, investigating the risk of falls in this group of people is particularly important, especially because post-operative or fracture management in patients with dementia is a challenge [35]. Furthermore, dementia is a strong risk factor for mortality after hip fractures and is associated with a high risk of entry into permanent care [36].

Several risk factors, including sex and medical diseases, are associated with falling events [13], but they were not significant in our study. Advancing age is a well-established risk factor for falls [13], and our results were congruent with those of previous studies [37,38,39]. However, other studies focusing on elderly individuals with cognitive impairment have found that age is not a significant risk factor for falls [40,41]. We propose that the impact of aging could be attenuated by other age-related comorbidities and the other aforementioned factors in this group of patients. Therefore, these results should be interpreted prudently. Sex differences are another demographic factor associated with the risk of falls, and previous studies have shown conflicting results: in the elderly, some studies found no significant association between sex and falling [41] and some studies found that the characteristics of falling differ significantly according to sex [39,42]. According to our findings, falling is quite complex. Comorbidities and sex may be associated with falls [13,14], but cognitive function, aging, mobility limitations, and daily and social activities might be more important.

This study had several limitations. First, it did not focus on a specific group but on a socially active population in the community. We included elderly individuals with normal cognitive function, questionable dementia, and mild dementia. Therefore, we could not perform subgroup analysis, because the subjects in our study were representative of the general population. However, this finding is still noteworthy for the general population. Second, we did not record medication and analysis, which are known risk factors for falls [11]. However, our participants were socially active and did not require assistance for activity, and the diversity of the characteristics of the enrolled subjects still provided useful results. Third, the impact and severity of falls were not evaluated in this study; therefore, further studies with longer follow-up periods are needed to evaluate post-fall complications.

## 5. Conclusions

In socially active elderly community dwellers, a history of falls, lower education levels, aging, mild dementia, and certain mobility limitations are risk factors for future falls. These results should be integrated into fall-prevention programs and health policies to reduce the damage caused by falls among people in an aging society.

## Figures and Tables

**Table 1:** General characteristics of community-dwelling elderly participants.

Variable	All (*n* = 232)	Falling Incidents in 1 Year		*p*-Value
		Falling Group (*n* = 64)	Non-Falling Group (*n* = 168)	
Age	70.5 ± 9.2	76.9 ± 7.5	68.0 ± 8.7	<0.001 *
Sex				0.127
Male	60 (26%)	12 (18.2%)	48 (28.6%)	
Female	172 (74%)	52 (81.3%)	120 (71.4%)	
Education				<0.001 *
≤6 year	81	40 (62.5%)	41 (24.4%)	
6–12 years	105	15 (23.4%)	90 (53.6%)	
<12 years	46	9 (14.1%)	37 (22.0%)	
Marital status				0.014 *
Married	135	29 (45.3%)	106 (63.1%)	
Widowed or single	97	35 (54.7%)	62 (36.9%)	
Exercise frequency				0.982
<3 per week	65	18 (28.1%)	47 (28.0%)	
≥3 per week	167	46 (71.9%)	121 (72.0%)	
History of falls				<0.001 *
Yes	66	46 (71.9%)	20 (11.9%)	
No	166	18 (28.1%)	148 (88.1%)	
Hypertension				0.220
Yes	101	32 (50%)	69 (41.1%)	
No	131	32 (50%)	99 (58.9%)	
Hyperlipidemia				0.878
Yes	45	12 (18.7%)	33 (19.6%)	
No	187	52 (81.3%)	135 (80.4%)	
Diabetes mellitus				0.783
Yes	48	14 (21.9%)	34 (20.2%)	
No	184	50 (78.1%)	134 (79.8%)	
Dementia (CDR)				0.012 *
0	120	24 (37.5%)	96 (57.1%)	
0.5 (questionable dementia)	98	33 (51.6%)	65 (38.7%)	
1 (mild dementia)	14	7 (10.9%)	7 (4.2%)	
Stroke				0.592
Yes	22	5 (7.8%)	17 (10.1%)	
No	210	59 (92.2%)	151 (89.9%)	
Anemia				0.483
Yes	14	5 (7.8%)	9 (5.4%)	
No	218	59 (92.2%)	159 (94.6%)	
MMSE	26.3 ± 4.2	24.7 ± 4.7	27.0 ± 3.8	0.001 *
Body mass index	24.5 ± 3.8	24.7 ± 4.1	24.3 ± 3.7	0.471

These results are expressed as the mean ± standard deviation or number (percentage). Note: A chi-squared test was used to compare categorical variables. The independent *t*-test was applied to compare the differences between groups. * *p* < 0.05. Abbreviations: CDR, clinical dementia rating; MMSE, mini-mental state examination.

**Table 2:** Differences in participants’ answers to mobility limitation questions associated with falling.

Mobility Item	All (*n* = 232)	Falling Incidents in 1 Year		*p*-Value
		Falling Group (*n* = 64)	Non-Falling Group (*n* = 168)	
Standing continuously for 15 min				0.003 *
No limitation	215	54 (84.5%)	161 (95.8%)	
Unable	17	10 (18.5)	7 (4.2%)	
Standing continuously for 2 h				0.001 *
No limitation	110	19 (29.7%)	91 (54.2%)	
Unable	122	45 (70.3%)	77 (45.8%)	
Sitting for 2 h				0.457
No limitation	185	49 (76.6%)	136 (81.0%)	
Unable	47	15 (23.4%)	32 (19.0%)	
Squatting				0.001 *
No limitation	128	24 (37.5%)	104 (61.9%)	
Unable	104	40 (62.5%)	64 (38.1%)	
Raising both hands over the head				0.248
No limitation	223	60 (93.8%)	163 (97.0%)	
Unable	9	4 (6.2%)	5 (3.0%)	
Grasping/turning objects with fingers				0.358
No limitation	216	58 (90.6%)	158 (94%)	
Unable	16	6 (9.4%)	10 (6%)	
Lifting 12 kg				0.025 *
No limitation	136	30 (46.9%)	106 (63.1%)	
Unable	96	34 (53.1%)	62 (36.9%)	
Walking 300 m				<0.001 *
No limitation	202	47 (73.4%)	155 (92.3%)	
Unable	30	17 (26.6%)	13 (7.7%)	
Climbing 3 flights of stairs				<0.001 *
No limitation	178	39 (60.9%)	139 (82.7%)	
Unable	54	25 (39.1%)	29 (17.3%)	
Running 30 m				<0.001 *
No limitation	130	20 (31.3%)	110 (65.5%)	
Unable	102	44 (68.7%)	58 (34.5%)	

Note: A chi-squared test was used to compare categorical variables. * *p* < 0.05.

**Table 3:** Binary logistic regression models of risk factors for falling over the course of 1 year.

Variable		B	S.E.	Wald	df	*p*	OR	(95% CI)
Age		0.162	0.034	23.163	1	0.000 *	1.176	1.101–1.256
MMSE		0.051	0.085	0.365	1	0.546	1.052	0.892–1.242
Marital status	Married	Reference						
	Widowed or single	0.645	0.488	1.743	1	0.187	1.905	0.732–4.962
Education	<12 years	Reference						
	6–12 years	−0.079	0.662	0.014	1	0.905	0.924	0.252–3.381
	≤6 years	1.408	0.682	4.267	1	0.039 *	4.088	1.075–15.549
Dementia (CDR)	0	Reference						
	0.5 (questionable dementia)	0.848	0.587	2.085	1	0.149	2.334	0.739–7.376
	1 (mild dementia)	3.032	1.384	4.800	1	0.028 *	20.729	1.376–312.195
History of falls		4.127	0.601	47.143	1	0.000	62.011	19.090–201.44
Standing continuously for 15 min		0.840	0.912	0.848	1	0.357	2.317	0.388–13.848
Standing continuously for 2 h		0.673	0.500	1.812	1	0.178	1.959	0.736–5.218
Squatting		0.331	0.480	0.476	1	0.490	1.393	0.543–3.569
Lifting 12 kg		0.267	0.481	0.307	1	0.579	1.305	0.509–3.351
Walking 300 m		1.424	0.658	4.683	1	0.030 *	4.153	1.144–15.077
Climbing 3 flights of stairs		0.932	0.523	3.177	1	0.075	2.539	0.911–7.076
Running 30 m		1.224	0.504	5.906	1	0.015 *	3.402	1.267–9.131

Note: Multiple binary logistic regression was used to determine the factors with significant differences, including age, marital status, dementia, and history of falls, and the significant differences from the mobility limitation questionnaire, including standing continuously for 15 min, standing continuously for 2 h, squatting, lifting 12 kg, walking 300 m, climbing three flights of stairs, and running 30 m, in all community elderly. Multiple binary logistic regression was performed by using the enter method and adjusted for age, MMSE, marital status, education, dementia, and history of falls. * *p* < 0.05. Abbreviations: CDR, clinical dementia rating; MMSE, mini-mental state examination.
